# Genetic Algorithm Workflow for Parameterization of a Water Model Using the Vashishta Force Field

**DOI:** 10.1021/acs.jpcb.4c06389

**Published:** 2025-01-21

**Authors:** Anthony Val C. Camposano, Even Marius Nordhagen, Henrik Andersen Sveinsson, Anders Malthe-So̷renssen

**Affiliations:** The Njord Centre, Department of Physics, University of Oslo, Sem Sælands vei 24, NO-0316 Oslo, Norway

## Abstract

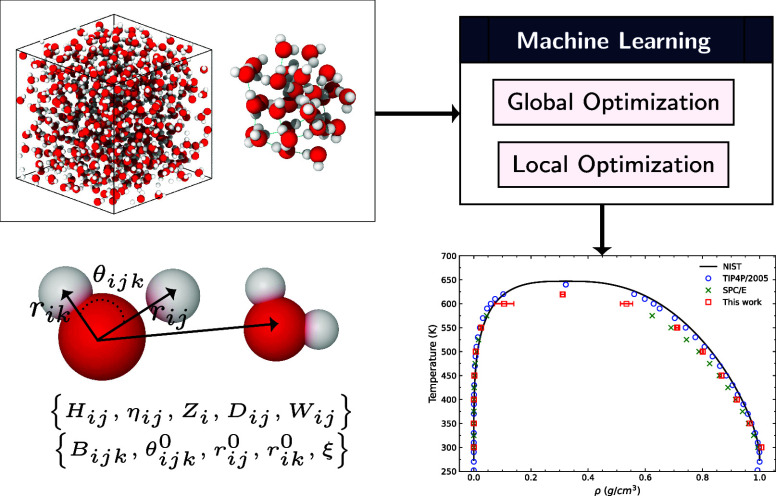

Water participates in countless processes on Earth, and the properties of mineral surfaces can be drastically changed in the presence of water. For example, the fracture toughness of silica glass is reduced by 25% for water-filled cracks than for dry cracks [*Geophys. Res.: Solid Earth***2018**, *123*, 9341–9354]. An accurate description of water is therefore essential for modeling the behavior of minerals in aqueous environments and, in particular, for modeling dynamic processes such as fracture, where the mechanical response of water may play an important role. On the molecular scale, molecular dynamics simulations with empirical force field methods provide a way to study large molecular systems at a relatively low computational cost. Many water models have been developed previously; however, a computationally cheap water model capable of describing reactions with minerals is lacking. Here, we present a parametrization of the water potential using the Vashishta potential form [*Phys. Rev. B***1990**, *41*, 12197–12209]. For this 3-point water model, we obtain good agreement with experimental transport and liquid–vapor properties. Importantly, the Vashishta form opens up compatibility with existing silica glass models, thus enabling the simulation of mineral–water interactions.

## Introduction

As two of the most abundant substances on Earth’s surface, water and silica have been extensively studied for decades both experimentally and computationally.^1−7^ However, certain properties of silica–water interactions remain elusive. In some environments, such as geological formations, the silica–water interface often encounters extreme conditions, such as high temperatures and pressures, which are difficult to replicate and study in a laboratory setting. Chemical reactions at the interface occur on extremely short time scales,^8,9^ making it challenging to observe the behavior using conventional experimental techniques.

Molecular dynamics simulations serve as a bridge to overcome these experimental limitations, providing powerful tools for investigating complex interfacial phenomena and complementing experimental studies by offering a microscopic understanding of silica–water interactions. For instance, molecular dynamic studies have been used to examine water confined within silica nanopores,^10−14^ which exhibit deviations in physicochemical properties compared to its bulk state. This has been subsequently confirmed by experimental studies, showing that confinement decreases the density and surface tension of water.^15^ Several simulations have shown water’s significant role in stress corrosion,^16^ crack tip propagation,^17^ and nanoscale friction^18^ studies. The presence of water facilitates fracture events by lowering the energy required for Si–O bond breakage. In tribochemistry, using a reactive water model, Yeon et al.^18^ showed that the amount of water present influences wear during sliding due to atom transfers involved in surface hydroxyl formation and water dissociation. The results have been shown to be consistent with experimental studies using AFM and macroscale ball-on-flat tribotests.^19,20^

Over the years, several water models have been parametrized to work with silica. To reduce computational costs, early water–silica interface modeling often employed rigid water models.^21−24^ However, these water models like TIP4P/2005, SPC/E, and TIP3P are nondissociating and therefore have inherent limitations in capturing the full complexity of interface behavior. For example, these models are unable to capture the process, where water dissociates and passivates freshly cut silica fracture surfaces. While more accurate water models^25,26^ incorporating many-body physics, polarization, and nuclear quantum effects have also been developed, their direct application to silica interfaces would require complex adaptations. The hydrolysis reaction, leading to silanol formation and water molecule dissociation, is a crucial aspect of silica–water interactions. Since this reaction involves chemisorption and proton transfer, a water model capable of dissociation and accurately describing its reactions with silica is highly desirable. Reactive interaction potentials like ReaxFF^27−29^ and the Mahadevan–Garofalini force field (MGFF)^30^ exist but come with high computational costs due to their focus on broad applicability and complex functional forms.

The Vashishta potential^31^ offers several distinct advantages for modeling water, especially when computational efficiency and the ability to simulate chemical reactions are priorities. Compared to rigid water models, which rely on constraint algorithms, such as RATTLE,^32^ SHAKE,^33^ and SETTLE,^34^ the Vashishta potential’s flexible three-body terms inherently allow bond breaking and formation without the need for additional constraints. This eliminates the computational overhead associated with such algorithms, leading to speedups. Furthermore, unlike other dissociative water models that utilize complex functions (e.g., error function, Gaussian charge distribution), the relatively simpler form of the Vashishta potential offers additional computational benefits. These advantages position the Vashishta potential form as a promising candidate for simulating large-scale systems involving water and its interactions with other materials, particularly in scenarios in which bond breaking and formation are crucial, such as at water–mineral interfaces.

In a study of cavitation by Shekhar et al.,^35^ the Vashishta potential was used to model reactive water in a billion-atom molecular dynamics simulation. The water model employed in that study has the same functional form as the established interatomic potential for silica,^31,36,37^ allowing seamless coupling through a bond-order scheme that accounts for the varying oxygen environment at the water–silica interface. This force field model has also been used to study nanoconfined water.^38^ For these studies, the force field parameters were optimized to reproduce the water structure (bond length and bond angle) and the structural properties of low-density and high-density supercooled water. However, this parametrization is unable to capture the transport and interfacial properties of water, which are important in studies of silica–water systems and therefore restricts its applicability. For example, in a dynamic fracture, the transport properties of water are important since this governs the speed of wetting on newly exposed crack surfaces.^39,40^ Surface tension and solid–fluid adhesion dictate water’s access to crack cavities; thus, accurately capturing its interfacial properties is also essential.

To address these issues, we introduce a new parameter set for the bond-ordered version of the Vashishta force field for water. Our parametrization is capable of reproducing key thermodynamic, interfacial, and transport properties of water over a wide range of temperatures and pressures. This parametrization may subsequently form the basis for reparameterization of the water–silica model of the Vashishta form.

## Methods

### Water Potential

In molecular dynamics simulations, we consider a system of *N* particles with positions *r*_1_, *r*_2_, ···, *r*_*N*_ and masses *m*_1_, *m*_2_, ···, *m*_*N*_. The potential energy of the system is a function of the position of all the particles: *U* = *U*({**r**}), and the force on a particle *i* is given by the negative gradient of that potential energy: **F**_*i*_ = −∇_**r**_*i*__*U*. This force is then integrated using Newton’s second law, typically through the Velocity Verlet scheme, to obtain updated velocities and positions of the particles.

A crucial aspect of molecular dynamics simulations is the selection of an accurate potential energy function. In this study, we model all interactions between the atoms of the water molecules using the following potential function^36,41^:
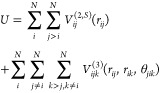
1The shifted two-body term *V*_*ij*_^(2,*S*)^ is evaluated for all pairs of atoms within a cutoff distance *r*_c_ and the three-body term *V*_*ijk*_^(3)^ is evaluated for all triplets of atoms within a cutoff *r*_0_.

The two-body term consists of steric repulsion, Coulomb interaction, charge–dipole interaction, and a term for the van der Waals interaction and is expressed by
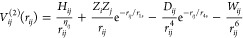
2Here, *r* ≤ *r*_c_, where *r*_*ij*_ = *r*_*i*_ – *r*_*j*_ is the distance between atoms *i* and *j*, *r*_1*s*_ is the screening length of the Coulomb interactions, *r*_4*s*_ is the screening length of the charge–dipole interaction, *H*_*ij*_ is the steric repulsion strength, *Z*_*i*_ is the effective charge of particle *i*, *D*_*ij*_ is the charge–dipole interaction strength, and *W*_*ij*_ is the van der Waals attraction strength, and η_*ij*_ is the exponent of the steric repulsion.

The two-body terms are shifted for *r* < *r*_c_ and truncated at the cutoff *r*_c_, making energy and forces at the cutoff continuous in the following way:
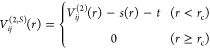
3where
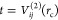
4
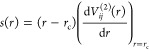
5

The three-body term consists of bending *f*(*r*_*ij*_, *r*_*ik*_) and stretching factors *p*(θ_*jik*_, θ_*jik*_^0^):
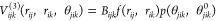
6where *B*_*ijk*_ modulates the strength of the three-body potential. The bending factor is defined as follows
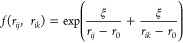
7for *r*_*ij*_, *r*_*ik*_ ≤ *r*_0_; otherwise, *f*(*r*_*ij*_, *r*_*ik*_) = 0. The stretching factor is given by
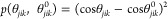
8Here, *r*_0_, ξ, and θ_*jik*_^0^ are free parameters, while θ_*jik*_ is the angle formed by the vectors *r*_*ij*_ and *r*_*ik*_, with atom *i* as the vertex. To limit the number of free parameters, the three-body term applies only to H–O–H triples, where *r*_0_ defines the cutoff value for the O–H bond length. Note that the angle θ_*jik*_^0^ is not directly the equilibrium angle of the water molecule due to two-body interactions between hydrogen atoms. The Vashishta potential’s three-body term is similar to the three-body expressions found in the work of Garofalini and Mahadevan^30^ and Hofer et al.^42,43^

Depending on the values of the free parameters, this potential function has the flexibility to model various substances. It has, for example, been parametrized for silica,^31^ silicon carbide,^36^ indium phosphide,^41^ and silica + water.^35^

### Genetic Algorithm Workflow

**Figure 1:**
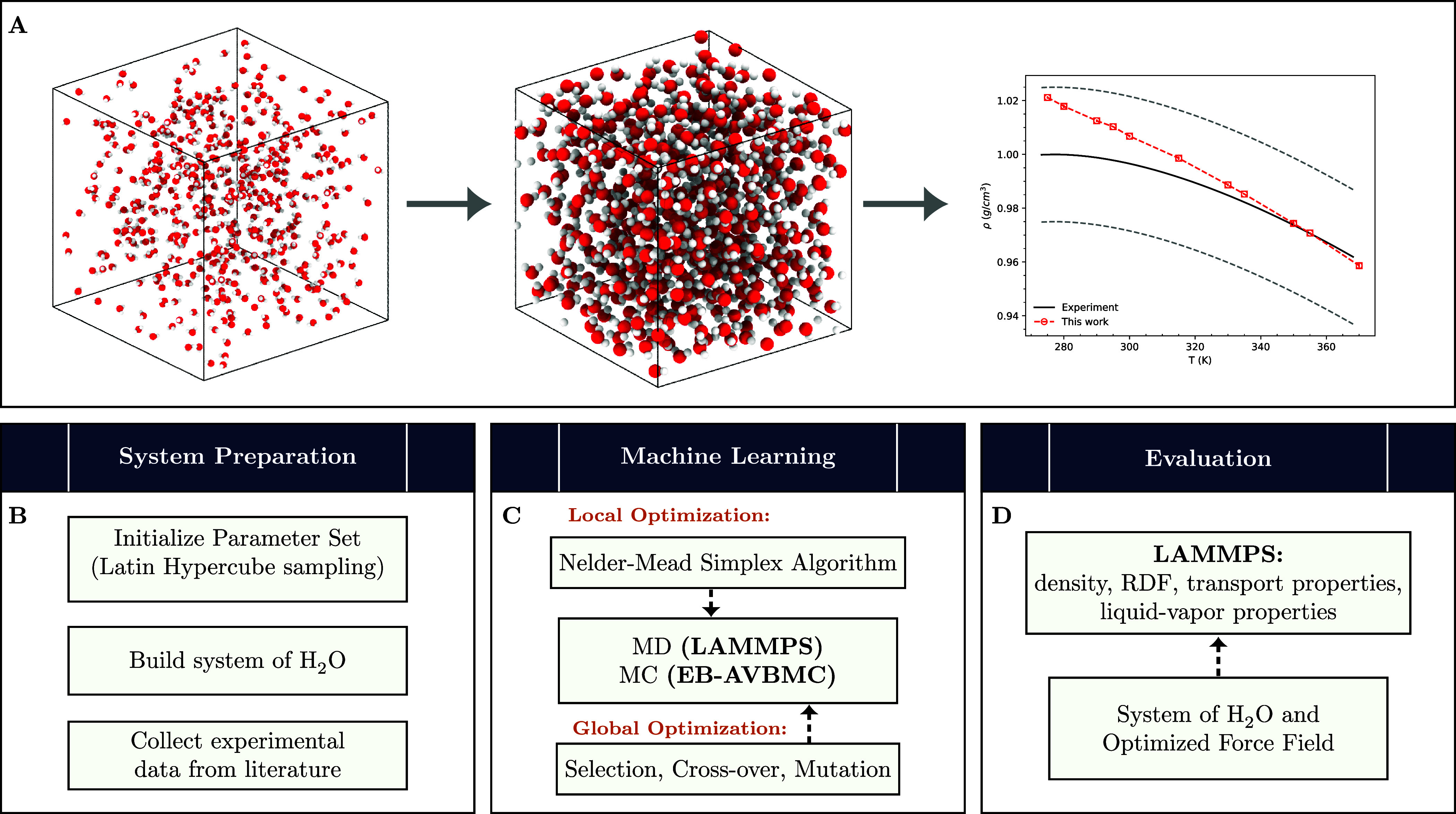
Overview of the force field optimization process with data preparation, training, and evaluation. (A) Parametrization process begins with a randomly generated parameter set, followed by iterative optimization to enhance accuracy, and concludes with evaluation based on the final parameters’ ability to reproduce key water properties. (B) Input data used for the training. (C) Force field parametrization scheme consists of a global optimizer (GA) to scan the parameter landscape, followed by local optimization (Nelder–Mead simplex algorithm) for refinement. (D) Evaluation of the best-performing parameter set is conducted by using it in simulations of thermodynamic conditions but not in the training set.

To optimize the parameters of the water model for multiple target properties simultaneously, we employ a hierarchical genetic algorithm (GA). The algorithm was used to iteratively improve model parameters to match key empirical target values, such as density, self-diffusion coefficient, radial distribution function (RDF), hydronium formation, gas density, and surface tension. Table 1 outlines the hierarchy and specific target values used in this study. Each parameter set is evaluated based on a hierarchical objective function, where its value measures its ability to reproduce a hierarchy of sets of properties defined by the user. The hierarchical objective genetic algorithm (HOGA)^44,45^ has been shown to be capable of accelerating the search in a high-dimensional parameter space. Following previous studies employing a HOGA algorithm,^44,45^ we first use a GA^46,47^ as a global optimizer, minimizing fitness over a user-specified number of iterations. This is then refined using the Nelder–Mead simplex algorithm^48^ for local optimization. Figure 1 outlines the overall methodology used in this study.

**Table 1:** Hierarchy of Target Properties

priority	thermodynamic condition	property name	tolerance
1	*T* = 298.15 K, *P* = 1 bar	density, ρ	
1	*T* = 298.15 K, *P* = 1 bar	self-diffusion, *D*	
1	*T* = 298.15 K, *P* = 1 bar	H_3_O^+^, OH^–^ count	
1	*T* = 298.15 K, *P* = 1 bar	RDF	
2	*T* = 298.15 K, *P* = 3008 bar	density, ρ	
2	*T* = 298.15 K, *P* = 3008 bar	self-diffusion, *D*	
3	*T* = 363.15 K, *P* = 1 bar	density, ρ	
3	*T* = 363.15 K, *P* = 1 bar	self-diffusion, *D*	
3	*T* = 363.15 K, *P* = 1 bar	H_3_O^+^, OH^–^ count	
4	*T* = 333.15 K, *P* = 2940 bar	density, ρ	
4	*T* = 333.15 K, *P* = 2940 bar	self-diffusion, *D*	
5	*T* = 273.15 K, *P* = 1 bar	density, ρ	
5	*T* = 273.15 K, *P* = 1 bar	self-diffusion, *D*	
5	*T* = 273.15 K, *P* = 1 bar	H_3_O^+^, OH^–^ count	
6	*T* = 283.15 K, *P* = 3032 bar	density, ρ	
6	*T* = 283.15 K, *P* = 3032 bar	self-diffusion, *D*	
7	*T* = 300 K	intercept of δΔ*G*	
7	*T* = 300 K	slope of δΔ*G*	
7	*T* = 373 K	intercept of δΔ*G*	
7	*T* = 373 K	slope of δΔ*G*	

With two particle types, oxygen (O) and hydrogen (H), the functional form of our water potential nominally contains 29 parameters. However, by restricting the three-body interactions to H–O–H and by fixing the two-particle cutoff distance at 5.5 Å, we are left with two parameters for the Coulomb interactions (*Z*_O_ = −2*Z*_H_ and *r*_1*s*_), four parameters for the single three-particle interaction (*B*_HOH_, θ_HOH_^0^, ξ and *r*_0_) and 15 non-Coulomb two-body parameters (η, *r*_4*s*_, *H*, *D*, and *W* for each of the two-body components H–H, H–O, and O–O). We fix three of the non-Coulombic parameters to zero (Table 2); hence, we are left with 18 free parameters to be adjusted by the HOGA algorithm.

**Table 2:** Two-Body and Three-Body Parameter Sets of the Vashishta Water Model

*Z*_O_ (e)	*Z*_H_ (e)	*r*_1*s*_ (Å)	*r*_c_ (Å)
–0.943117	0.471558	4.477392	5.5

	H–H	H–O	O–O
η_*ij*_	7.391415	13.237354	7.617388
*r*_4*s*_ (Å)	1.948928	1.661659	1.965379
*H*_*ij*_ (eV Å^η^)	0.0	0.450836	1232.69421
*D*_*ij*_ (eV Å^4^)	0.0	1.428122	91.090399
*W*_*ij*_ (eV Å^6^)	0.0	0.229314	10.599231

	*B*_*ijk*_ (eV)	θ_0_ (°)	ξ (Å)	*r*_0_ (Å)
H–O–H	259.861109	100.147683	0.503514	1.232741
O–H–O	0	0	0	0

From pilot runs of the algorithm, we found that the performance of the global optimization algorithm was more sensitive to the allowed range of the parameters of the force field than on the specific details of GA tuning.^49^ Therefore, we carefully created an initial parameter set (see Table S1) by looking at an existing empirical potential^43,50^ that has an almost identical functional form to that of the Vashishta force field. For the two-body interaction, we use curve fitting to match the Vashishta potential’s non-Coulombic and Coulombic parameter sets to that of an existing potential within the truncation range of 5.5 Å separately. For the initial three-body parameter set, we use the values provided in the parameter set of Hofer and Wiedemair.^43^ The initial population of size *P*–1 consists of randomly generated parameters within a user-specified range (see Table S2) around the initial parameters, using Latin hypercube sampling.^51^

The GA works by initializing a population of solutions generated by varying around the initial parameter set. This population evolves over multiple generations, where each candidate solution is evaluated for its fitness, and the best-performing solutions are selected for reproduction. The algorithm’s main operations—selection, crossover, and mutation—facilitate the exploration of the parameter space and the optimization of the solutions.

This study employed a GA with pool-based selection.^52^ Tournament selection (without replacement) was used to choose between 2 random parameter sets from the population. Mutation and crossover operations were applied to the best-performing parameter sets to generate new ones. For the crossover operations, a simulated binary operation was used. For the mutation operation, a polynomial mutation was used with a polynomial order of 20 and a mutation rate of 30%. A population size of *P* = 40 was set for each iteration of the GA that evolved for 200 generations. The best individual of the iteration serves as the base parameter for the next iteration of the GA simulations, and this process was repeated five times. After the convergence criteria of the global optimization were satisfied, a Nelder–Mead simplex algorithm was used to refine the parameter set. The input of the simplex algorithm was the best result of the GA and is randomly perturbed to generate a *P* + 1 parameter set. The best parameter set was chosen based on its overall performance in the validation tests.

### Target Values and Calculation Methods

The training targets we use in the HOGA algorithm are mass density, self-diffusion, H_3_O^+^ and OH^–^ concentrations, RDF, and surface tension of the water–vapor interface. We optimize these targets according to the hierarchy listed in Table 1. The target properties are all empirical values from the experiments. The gas densities, liquid densities, and surface tensions are obtained from the NIST database.^53^ The self-diffusion coefficients were obtained from Easteal et al.^54^ We used 3 training points at temperatures *T* = 273.16, 298, and 363.15 K using pressure *P* = 1 bar and another set of training points at high-pressure values at *P* = 2940, 3008, and 3032 bar for temperature *T* = 363.15, 298.15, and 283.15 K, respectively. In addition to the density and self-diffusion under ambient conditions, we include training points at high pressure and high temperature to capture a wider range of liquid and gas phases. We also included saturated vapor gas densities and surface tension at temperatures *T* = 300 K and *T* = 373 K in the hierarchy to capture the interfacial characteristics of water.

Running the HOGA algorithm to optimize a force field requires calculating the target properties given a set of force field parameters and comparing them to empirical target values. To compute the target values of the HOGA algorithm, we ran the following set of simulations:

- Molecular dynamics simulation of 512 water molecules in the liquid state, running in the NPT ensemble for 100 ps (equilibration), followed by 150 ps in the NVT ensemble.
- Molecular dynamics simulation of 10,000 water molecules in the liquid state, NPT ensemble, 50 ps (only for H_3_O^+^ concentration).
- Energy-biased aggregation volume-biased Monte Carlo sampling.

To evaluate the model’s performance, we utilize larger systems (1024 and 10^5^ water molecules) and longer simulation times (1 ns NPT, then 5 ns NVT, and 100 ps for hydronium calculation) than those used in training. We also employ direct coexistence molecular dynamics simulations for liquid–vapor properties instead of EB-AVBMC. Additional simulation details are provided in subsequent sections.

### Calculation of Water Model Properties

The molecular dynamics simulations were performed using LAMMPS (17 Feb 2022)^55^ using the pair-style pair_style vashishta. The temperature and pressure were kept constant using a Nosé–Hoover^56,57^ thermostat and barostat.

From these molecular dynamics simulations, we sampled the following properties: density, RDF, transport properties, hydronium formations, and liquid–vapor properties, as described below.

### Density and Radial Distribution Function

The density of the water system is sampled from the last 50 ps of the NPT molecular dynamic simulation. This is followed by an NVT ensemble simulation with the volume set to the average volume computed from the NPT ensemble to obtain additional properties such as the RDF and the transport properties.

### Transport Properties

For transport property calculation, we used an existing LAMMPS plugin called OCTP^58^ (on-the-fly calculation of transport properties) for efficiency. Initially, a molecular dynamics simulation in the isothermal–isobaric ensemble was performed to determine the equilibrium density of the system, followed by a canonical ensemble simulation using the average volume from the isothermal–isobaric ensemble simulation to sample self-diffusion and viscosity.

### Self-Diffusion Coefficient

The transport coefficients were computed from the Einstein relation, and for efficient sampling of mean-squared displacement (MSD), an order-n algorithm^59^ was used. The self-diffusion coefficient was computed using the following expression:
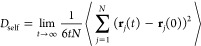
9where *N* is the number of water molecules, *t* is the correlation time, and **r**_*j*_ is the position of water molecule *j*. During the canonical ensemble simulations, the viscosity was also computed. The viscosity was used for the finite-size effect correction of the self-diffusion coefficient. Finite-size effects must be taken into account when calculating the self-diffusion coefficient; otherwise, the values will be underestimated.^60^ Yeh and Hummer^61^ derived a size-dependency relationship between self-diffusivity computed from molecular dynamics simulations and self-diffusion at the thermodynamic limit (*L* = ∞). The self-diffusivity of liquid water at the thermodynamic limit *D*_∞_ is given by
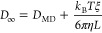
10where *D*_MD_ and η denote the self-diffusivity and shear viscosity obtained from the simulation, respectively. Here, *T* is the temperature, *L* is the length of the system, ξ = 2.837297 is a dimensionless constant, and *k*_B_ is the Boltzmann constant. For training and evaluation of the self-diffusion in this work, we incorporated this finite-size correction.

### Shear Viscosity

The shear viscosity was calculated using the following equation:
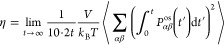
11where the traceless pressure tensor *P*_αβ_^os^ is defined as
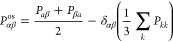
12and (*V*, *T*) is the volume and temperature of the system, *k*_B_ is the Boltzmann constant, δ_αβ_ is the Kronecker delta, and *P*_αβ_ are components of the pressure tensor.

### Liquid–Vapor Equilibrium Properties

To perform direct coexistence interfacial simulations, we use a parallelepiped rectangular cell with volume *V* = *L*_*x*_*L*_*y*_*L*_*z*_, where *L*_*x*_ = *L*_*y*_ = *L*_*z*_/5. The *L*_*x*_ dimension is determined using the system’s average water molecule density. Following Muller et al.,^62^ this average density is calculated as ρ = (ρ_L_ + ρ_V_)/2, where ρ_V_ and ρ_L_ denote the experimental liquid and vapor densities of water, respectively. Given the desired number of molecules (*n*) and the average density (ρ), the total volume is calculated using *V* = *n*/ρ, and subsequently, *L*_*x*_ = (*V*/5)^1/3^. Periodic boundary conditions are imposed; hence, two independent interfaces are formed, bounding the bulk phase.

To simulate the interfacial properties, we employed the temperature-quench molecular dynamic simulation (TQMD) method.^63^ All simulations were conducted using a constant volume and temperature with a thermostatic damping parameter of 50 fs. The initial configuration is homogenized at a high temperature (700 K) for 25 ps. After that, the temperature of the simulation is abruptly scaled to the desired temperature for 1 ns, followed by a 4 ns production stage for each temperature between 300 and 450 K. For temperatures between 500 and 600 K, a longer 6 ns production stage was used. Liquid and vapor densities were obtained from the density profile along the *z*-direction, utilizing the last 2 ns of the simulation (or the last 0.5 ns for *T* = 600 K).

### Density and Critical Parameters

We obtained the liquid-phase densities ρ_l_ by fitting the density profile along the *z*-direction to the hyperbolic tangent-based equation:
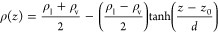
13where *z*_0_ is the position of the interface and *d* is the interfacial thickness. Vapor-phase densities ρ_*v*_ are calculated by averaging the density profile within the vapor region, ensuring a distance of at least 10 Å from the vapor–liquid interface for each temperature between 400 and 600 K and at least 2 Å for each temperature between 300 and 350 K.

To determine the critical density ρ_c_ and temperature *T*_c_, we fit the coexistence densities (ρ_l_, ρ_v_) for temperature between 450 and 600 K to the law of rectilinear diameters and the universal scaling of the coexistence densities, which are described by the following set of equations:
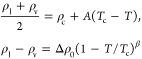
14where β = 0.326 is the Ising model critical exponent. The parameters *A* and Δρ_0_ are fitting parameters dependent on the simulated system.

### Surface Tension

We calculated the surface tension γ using the diagonal components of the pressure tensor obtained from direct coexistence simulations. The following equation was employed:
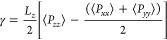
15where *L*_*z*_ is the box length in the *z*-dimension and *P*_*xx*_, *P*_*yy*_, and *P*_*zz*_ are the diagonal components of the pressure tensor. The pressure tensor components were recorded during the production phase of the simulation, and the surface tension was calculated by using the average of these recorded pressure values.

### Enthalpy of Vaporization and Vapor Pressure

The procedure we used follows the one outlined by Muniz et al.^64^ We conducted separate canonical ensemble simulations for the liquid and vapor phases using densities obtained from our direct coexistence simulation at each respective temperature. We used 512 water molecules for the vapor phase and 1024 water molecules for the liquid phase. The thermostat damping parameter of 50 fs and a time step size of 0.5 fs were used in the constant volume and temperature simulation. The enthalpy of vaporization was calculated using
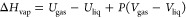
16where we use the gas-phase pressure *P*, volume (*V*_liq_, *V*_gas_), and total energy (*U*_gas_, *U*_liq_) from the canonical simulations. Saturation vapor pressure was also obtained from the canonical simulations for temperatures ranging from 300 to 600 K.

### Boiling Point and Critical Pressure

We fitted the saturation vapor pressure data to the Antoine equation^65,66^:
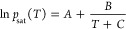
17where *A*, *B*, and *C* are constant parameters, *T* is the temperature, and *p*_sat_ is the saturated vapor pressure. This allowed us to calculate the boiling point *T*_b_ at *P* = 1.013 bar and the critical pressure *P*_c_ using the previously determined critical temperature *T*_c_.

### Water Self-Ionization

Water self-ionization is known to be a rare event.^67,68^ While accurately computing its frequency during HOGA optimization would require prohibitively long or large simulations, we can use the rarity to set a reasonable constraint on our model. We require that ionization events occur with a frequency of less than 1/10^5^. In our system of 10^5^ water molecules, this corresponds to at most one dissociated water molecule on average through a simulation.

### EB-AVBMC Simulations

A fast estimate of surface tension and gas density can be achieved by calculating the free energies of water clusters. For this case, we use the established energy-biased aggregation volume bias Monte Carlo (EB-AVBMC)^44,69−71^ technique. To perform energy-bias aggregate Monte Carlo simulations, we extended the code of Loeffler et al.^69^ to include the interatomic potential model described by eqs 3 and 6. We modified the insertion and deletion schemes to take into account the reactive nature of our water potential. This allows us to include the vapor–liquid properties of water in our parametrization, taking advantage of classical nucleation theory,^72^ which relates the distribution of cluster size to the free energy.

The classical nucleation theory equation, where the slope and intercept are used for training, is given by the following equation:
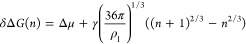
18where ρ_l_ is the liquid-phase density, γ is the surface tension, and Δμ is the chemical potential. The chemical potential is related to the gas density by
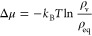
19where ρ_v_ is the user-defined gas density and ρ_eq_ is the equilibrium vapor density, *T* is the temperature, and *k*_B_ is the Boltzmann constant. The slope and intercept were obtained from a linear fit of δΔ*G* with the cluster size ((*n* + 1)^2/3^ – *n*^2/3^). By fitting the slope and intercept to the expected values, we can obtain a good estimate of surface tension and vapor gas density. We used 1.5 million cycles, where each cycle has 100 moves, to obtain a good approximation of the slope and intercept. Moves in the Monte Carlo simulations consist of single-atom translations and an AVBMC swap move to mimic the destruction and creation of bonds. The Stillinger cluster criterion^73^ distance used was 4 Å. The deletion move is constrained to a water molecule with two hydrogen atoms that are within a distance of 2 Å from oxygen. We used a maximum cluster size of 30 water molecules and a gas number density of 6 × 10^–7^ Å^–3^.

## Results and Discussion

### Trained Water Model

The optimized parameter set after running 200 generations of GAs with a population size of 40 and a refinement using 50 cycles of the Nelder–Mead simplex algorithm is listed in Table 2. For the evaluation of the performance of the model, we use a larger system and a longer simulation time. We use a system consisting of 1024 water molecules for transport and thermodynamic property simulations. For the assessment of water reactivity, we use a larger system consisting of a hundred thousand water molecules. The performance of the model is characterized through its ability to reproduce the transport and liquid–vapor properties over a wide range of temperatures and pressures.

### Temperature Dependence of Density and Self-Diffusion Coefficient

**Figure 2:**
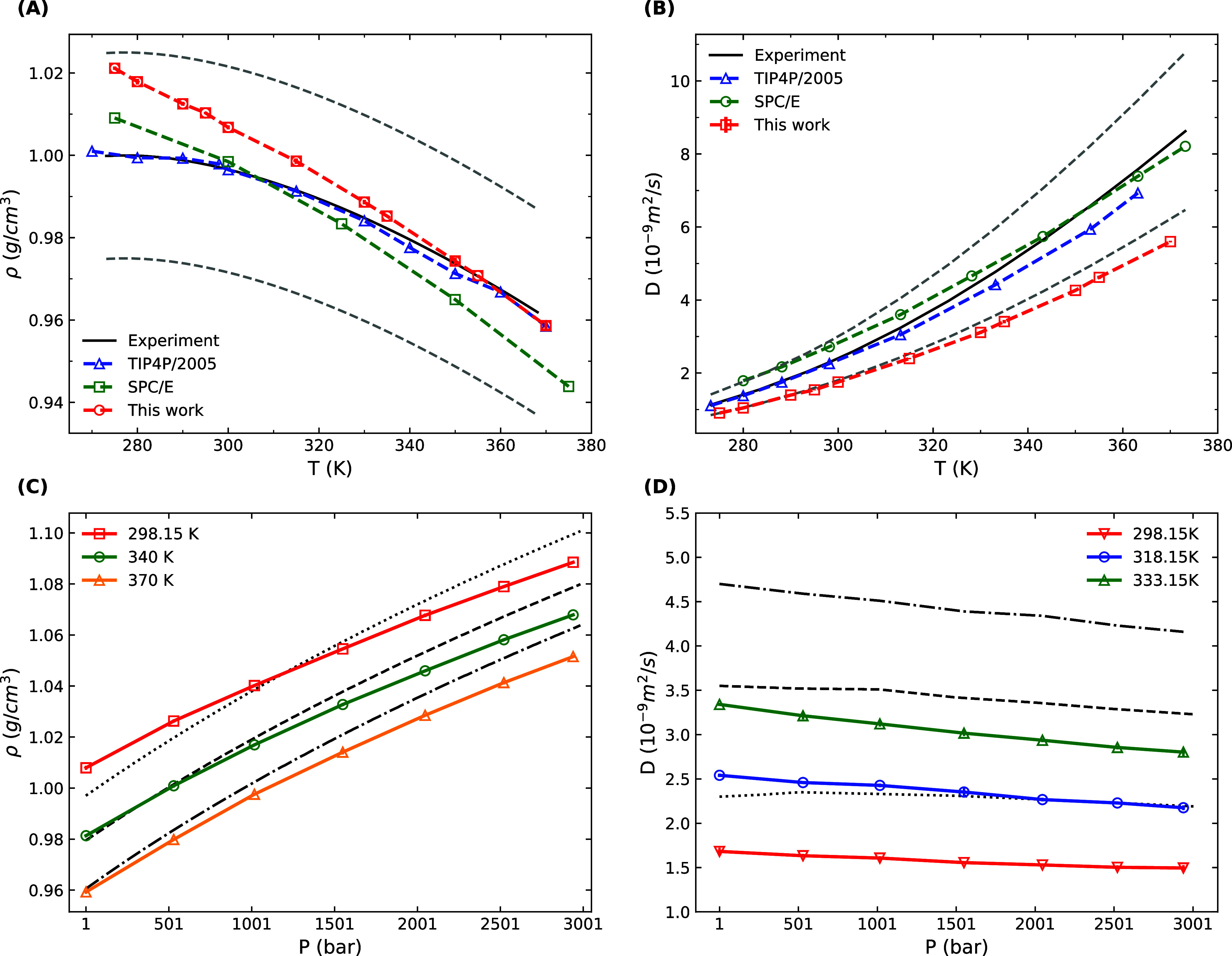
Temperature and pressure dependence of density and self-diffusion coefficient of water. (A) Density–temperature curve of liquid water at a pressure of 1 bar. Experimental,^53^ TIP4P/2005,^74^ and SPC/E^75^ data are shown for comparison. (B) Self-diffusion coefficient at a pressure of 1 bar. Experimental values of the self-diffusion (solid line) were obtained from Easteal et al.^54^ Two commonly used water models for mineral–water simulations, TIP4P/2005^76^ and SPC/E,^76^ are shown as well. The gray dashed lines in panels (A) and (B) are the limits of the acceptable values during the fitting process. (C) Liquid density of water as a function of pressure. Solid lines are liquid densities obtained from the MD simulation. Experimental values of temperature at 298 K (dotted), 340 K (dashed), and 370 K (dash-dot) are obtained from NIST.^53^ (D) Self-diffusion coefficient as a function of pressure. Experimental values^77^ of the self-diffusion for temperatures 298.15 K (dotted), 318.15 K (dashed), and 333.15 K (dash-dot) are given as references.

We run simulations at 12 different temperatures at constant pressure to evaluate the ability of the water potential to reproduce the thermal expansion curve. For comparison, the density and self-diffusion coefficient results from SPC/E and TIP4P/2005 are also shown in Figure 2A,B. The density and self-diffusion coefficients of our water model at a temperature between 280 and 370 K and a pressure of 1 bar are found to be within acceptable accuracy compared to the experimental results shown in Figure 2A,B and are on par with existing nonreactive water models commonly used in silica–water simulations. At 300 K and 1 bar, the calculated density from our water model is ρ = 1.01 g cm^–3^ with a relative error of 1% from the experimental value of ρ = 0.997 g cm^–3^. The calculated self-diffusion coefficient is *D* = 1.75 × 10^–9^ m s^–2^ at 300 K, which is close to the experimental result^54^ of *D* = 2.30 × 10^–9^ m s^–2^at 298.15 K. The deviations from experimental values for the densities are within the tolerance that we set during the fitting of the potential. However, the deviations in self-diffusion coefficients were adjusted during the Nelder–Mead optimization and, as a result, do not conform to the user-defined tolerance. This adjustment, however, enhanced the performance of other properties.

### Pressure Dependence of Density and Self-Diffusion Coefficients

In the field of geochemistry, the behavior of water at a high pressure is very important. Thus, we also evaluated the performance of the parameter set at a higher pressure. We evaluated the density and self-diffusion coefficients at temperatures of 298.15, 340, and 370 K applying 7 isotropic pressures between 1 and 3000 bar. It was observed that the computed values were lower than the experimental results as the pressure increases. Although our water model is less compressible compared to real water, the qualitative trend of density at three different temperatures over a wide pressure range shown in Figure 2C follows the experimental values with a maximum deviation of 2.5%, which is within the tolerance we chose during the fitting. Figure 2D, similarly, shows that the self-diffusion coefficients were underestimated as the pressure increased. However, our water model has better performance at higher pressure than the SPC rigid water model commonly used in ClayFF^21^ for a self-diffusion coefficient between 1 bar and 3000 bar.^78^

### Shear Viscosity

**Figure 3:**
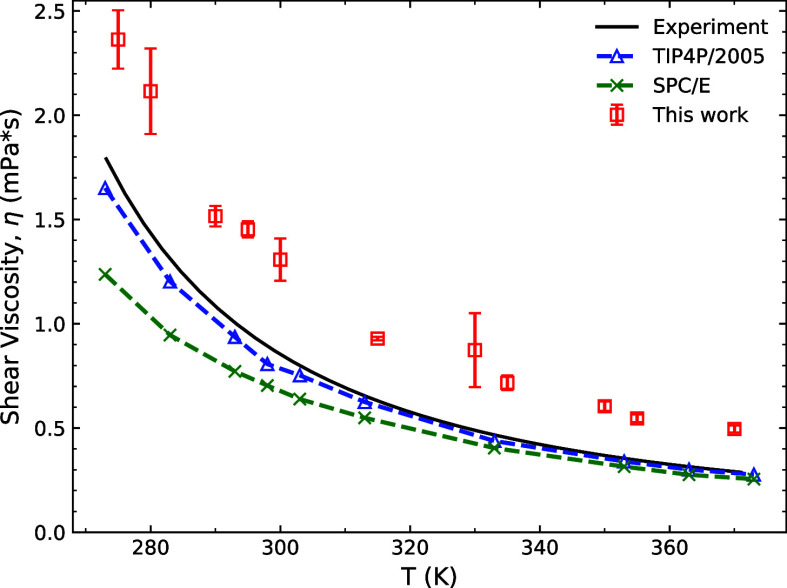
Shear viscosities of water obtained from a molecular dynamic simulation of 1024 water molecules. Molecular dynamic simulation results for shear viscosity are overestimated compared with the experimental values. The SPC/E and TIP4P/2005 results^80^ are provided for comparison. The experimental data^53^ are shown in a solid line. Error bars represent one standard deviation based on five different measurements.

Accurate modeling of water’s viscosity is crucial in simulations that involve compression and shearing forces within fluids. Figure 3 shows the shear viscosities at *T* = 275–370 K obtained from our molecular dynamics simulation. Shear viscosity values follow the experimental trend. At a temperature of 300 K and pressure of 1 bar, the calculated shear viscosity is η = 1.30 (10) mPa s, showing a deviation of 51% from the experimental value of 0.85 mPa s. We expect a similar quality of accuracy for shear viscosity since we fitted the water model to a set of experimental values of the self-diffusion coefficient. The self-diffusion coefficient is underestimated; thus, the shear viscosity is overestimated. These observations follow the Stokes–Einstein relation.^79^

### Vapor–Liquid Equilibrium Properties

**Figure 4:**
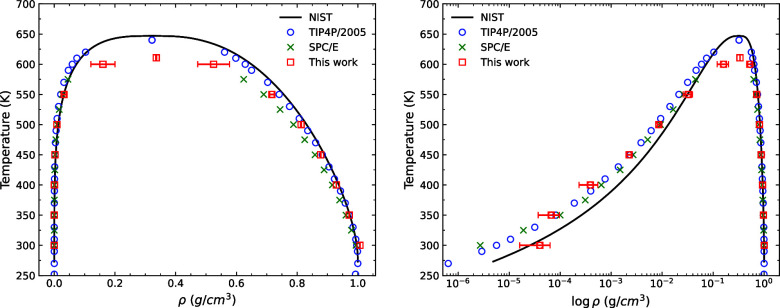
Vapor–liquid coexistence curve of the Vashishta water potential compared to experimental^84^ values, SPC/E^85^ and TIP4P/2005.^86^ The data on the right is in a log scale to show the vapor-phase density trend. Error bars represent one standard deviation based on 5 different measurements.

**Figure 5:**
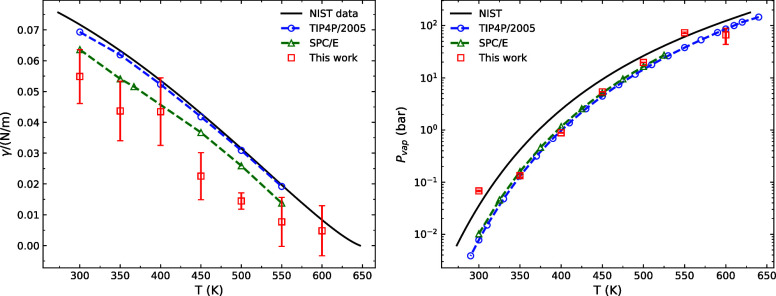
(Left) Surface tension of water using the Vashishta potential compared to TIP4P/2005 and SPC/E from the work of Vega and de Miguel.^87^ (Right) Saturation vapor pressure for SPC/E^88^ and TIP4P/2005^86^ compared with the result of this work. Error bars represent one standard deviation based on 5 different measurements.

An important test of the quality of the water potential is its ability to reproduce different phases of water. One way is to do an interfacial simulation to reproduce the vapor–liquid coexistence curve. Despite a lack of explicit long-range interactions, our optimized water potential is capable of reproducing the coexistence curve (Figure 4), surface tension, and vapor pressure (Figure 5) within an acceptable accuracy. This finding is consistent with the results reported by Yue et al.^82^ The critical parameters and enthalpy of vaporization shown in Table 3 are on par with the SPC/E values, a 3-point rigid water model commonly used in mineral–water simulations.^24,83^

**Table 3:** Thermodynamic Properties of the Vashishta Water Model

property	experiment^53^	this work	SPC/E^81^
*T*_c_ (K)	647.1	611	623
ρ_c_ (g/cm^3^)	0.322	0.336	0.273
*T*_boil_(K)	373	396	402
*P*_critical_ (bar)	220.64	141	134
Δ*H*_vap_ (kcal/mol)	10.51	12.14	11.99

### Surface Tension

Using the direct coexistence method, we also obtain the vapor–liquid surface tension using the pressure tensor calculated from eq 15 for temperatures between 300 and 600 K. As expected, the surface tension between the temperatures 300 and 370 K is close to the experimental values since we explicitly targeted these during fitting using the EB-AVBMC simulations. The trend of our model’s surface tension also follows the experimental curve at higher temperatures above 368 K. At 300 K, the surface tension of our parametrization is γ = 54 ± (8) mN m^–1^, close to the values of TIP3P and SPC,^89^ which are 49.5 and 53.4 mN m^–1^, respectively. The experimental value of the surface tension at the same temperature is 72 mN m^–1^. To our knowledge, this is the first parametrization of the Vashishta potential for water that captures an acceptable surface tension. Previous Vashishta potential for water has not been parametrized for water’s interfacial properties, making it unsuitable in interface modeling, such as wetting phenomena on surfaces, droplets on a hydrophobic interface, or bubble formation.

### Vapor Pressure

The vapor pressure obtained from our water model follows the trend of the experimental results and is on par with the results of both the TIP4P/2005 and SPC/E values, as shown in Figure 5. The vapor pressures obtained are lower than the experimental vapor pressures, hence leading to an overestimation of the boiling point by 23 K. However, it performs slightly better than SPC/E in this regard.

### Water Reactivity

**Figure 6:**
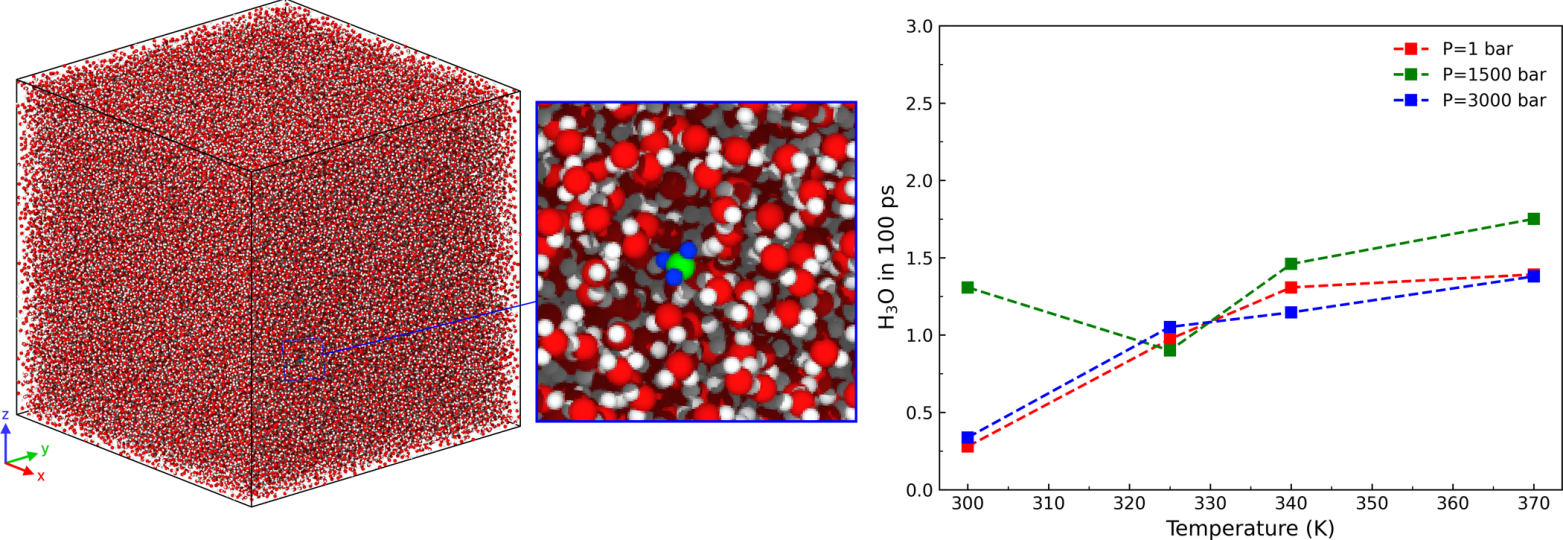
(Left) Water dissociation analysis was carried out in a system consisting of a hundred thousand water molecules. The inset shows a hydronium molecule (green represents the oxygen and blue represents the hydrogen) formed on the first 100 ps of an isobaric–isothermal ensemble at a temperature of 340 K at 1 bar. (Right) Amount of hydronium formed over a span of 100 ps increases with temperature but still in a negligible amount.

To count the number of autodissociation events in the system, we measured the number of hydronium formations at each time step. We determine the optimal value of the bond order cutoff by counting the number of OH^–^ and H_3_O^+^ in the system and selecting the cutoff that gives an equal number of both ions. The bond cutoff used in calculating the relative concentration of OH^–^ and H_3_O^+^ is 1.2 Å throughout the simulation time. We run three simulations for each temperature (300, 325, 340, 370 K) and pressure (1, 1500, 3000 bar) to see how reactive our water model is. We then measure the average amount of hydronium in the water for the first 100 ps. Hydronium formation, as shown in Figure 6, occurs rarely, even in a large system.

## Conclusions

Using the HOGA, we have demonstrated the capability of the Vashishta potential to represent the properties of water over a wide range of temperatures and pressures. The Vashishta potential has been applied in various molecular systems, such as silicon carbide,^36^ aluminum oxide,^37^ indium phosphide,^41^ and silicon dioxide.^31,90,91^ It has also been shown in previous studies^35,38^ that the same functional form can be used to represent the atomic interaction of the water system at a certain thermodynamic condition. Here, we have shown the ability of the Vashishta potential to reproduce the thermodynamic and interfacial properties of water. Our evaluation results show that the parametrized Vashishta potential for water exhibits transport and liquid–vapor properties on par with those of existing 3-site and 4-site rigid water models. This suggests that the Vashishta water model can be utilized for simulating a silica–water interfacial system, allowing for hydroxyl formation and water dissociation on the silica surface, instead of relying on a rigid water model. This dissociative water force field can be integrated into the existing silica potential to study processes such as dissolution, friction, and stress corrosion fracture over a wide range of thermodynamic conditions for larger-scale and longer-scale simulations. The parametrization for the silica–water interface will be presented in a later work.

Our newly parametrized dissociative water potential is already available in LAMMPS since it only involved supplying a new parameter file to pair-style vashishta from the MANYBODY package. Input files follow the standard Vashishta potential format, and for convenience, we supply a parameter file in the Supplementary Data. This parameter set can be refined and reparametrized to perform better on a specific target property by continuing the HOGA algorithm with additional properties in the hierarchy. The training procedure that we presented can be utilized to further extend the ability of the water potential in this study. Further extensions to the calculation of properties can include the dielectric constant, fluctuation-related properties, and some liquid–solid properties. Following the work of Wiedemair et al.,^92^ the inclusion of proton dynamics represents a promising avenue for future refinement of the parametrization. While the short-range nature of our force field may impose some limitations on reproducing certain thermodynamic^82^ properties, it offers a valuable trade-off for enhanced computational efficiency, particularly in large-scale and long-time-scale simulations.

## Supporting Information Available

Force field parameters used in the study (TXT)
Radial distribution function (RDF) of the water model; peak values for fitting; and experimental data of surface tension and gas density used in Monte Carlo simulation (PDF)
Molecular dynamic trajectory of water at 300 K and 1 bar (MP4)
Molecular dynamic trajectory of water cluster at 300 K in a microcanonical ensemble simulation (MP4)
