# Supplementary material for: Genetic Algorithm Workflow for Parameterization of a Water Model Using the Vashishta Force Field
Source: J Phys Chem B. 2025 Jan 21;129(4):1331–42. doi: 10.1021/acs.jpcb.4c06389 (PMC11789154; doi:10.1021/acs.jpcb.4c06389)
Supplement: Supplementary file 2 — jp4c06389_si_002.pdf [file jp4c06389_si_002.pdf]

Supporting Information: A Genetic Algorithm Workflow for Parameterization of a Water Model Using the Vashishta Force Field

Anthony Val C. Camposano, Even Marius Nordhagen, Henrik Andersen Sveinsson, and Anders Malthe-Sørenssen*

The Njord Centre, Department of Physics, University of Oslo, Sem Sælands vei 24, NO-0316, Oslo, Norway

E-mail: malthe@fys.uio.no

Phone: +47 22856477

Initial Parameter Set

Table S1: Initial two-body parameters obtain from curve-fitting and three-body parameters obtain from Hofer and Wiedemair.[S1]

Z_O (e)	Z_H (e)	r_{1s} (Å)	r_c (Å)
-0.901803	0.45090	3.1100	5.5

	H-H	H-O	O-O
η_{ij}	7.82	12	7.82
r_{4s} (Å)	0.00053	1.207319	0.00053
H_{ij} (eV Å$^\eta$)	0.0	0.2895693	1300.902
D_{ij} (eV Å^4)	0.0	0.050268	3.463888
W_{ij} (eV Å^6)	0.0	0.0311492	156.9247

	B_{ijk} (eV)	θ_0 (deg)	ξ (Å)	r_0 (Å)
H-O-H	122.4435	98.09	1.173	1.54
O-H-O	0	0	0	0

Ranges of the Initial Parameter Set

Table S2: The ranges of the two-body and three-body parameter values used in the Latin hypercube sampling to generate the initial population.

Z_O (e)	r_{1s} (Å)	r_c (Å)
-0.9 - -0.8	2.0-4.50	5.5

	H-H	H-O	O-O
η_{ij}	7.1–7.7	11.0–13.9	7.1–7.7
r_{4s} (Å)	0.001–2.0	1.90–2.0	0.001–2.0
H_{ij} (eV Å$^\eta$)	0.0	0.20–0.55	1295–1305
D_{ij} (eV Å^4)	0.0	0.003–1.5	0.0–132
W_{ij} (eV Å^6)	0.0	0.0–0.05	150–160

	B_{ijk} (eV)	θ_0 (deg)	ξ (Å)	r_0 (Å)
H-O-H	50-150	95-100	0.5-1.3	1.3-1.6
O-H-O	0	0	0	0

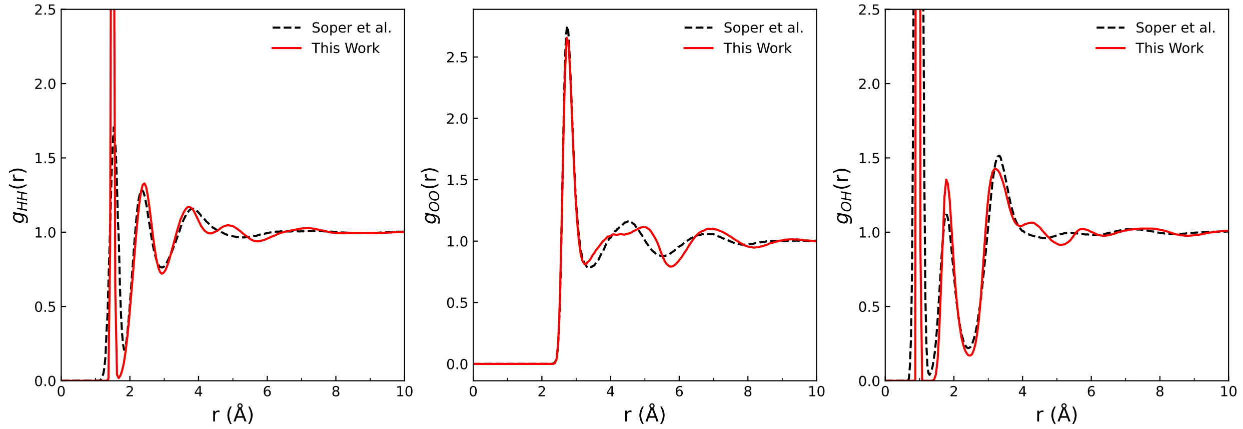

Figure S1: Radial distribution function of water at 300 K and 1 bar.

Radial Distribution Function (RDF)

We parameterized our dissociative potential to the 1st highest peak and lowest trough using experimental values given in Table S3 and included the 2nd highest peak and lowest trough for O-O pair.

Table S3: The values used in the fitting. The values are obtained from the work of Soper.[S2]

Pair	Position (Å)	Height
O-O 1st highest peak	2.73	2.75
O-O 1st lowest trough	3.36	0.78
O-O 2nd highest peak	4.26	1.11
H-H 1st highest peak	1.53	1.71
H-H 1st lowest trough	1.83	0.21
O-H 1st highest peak	1.29	2.75
O-H 1st lowest trough	3.36	0.04

EB-AVBMC Simulation

To obtain a good estimate of the surface tension and gas density, we followed the same procedure by Loeffler et al.[S3] in using the EB-AVBMC simulation results to fit the following equation,

$$\delta \Delta G(n) = \Delta \mu + \gamma \left(\frac{36\pi}{\rho_{liq}} \right)^{1/3} ((n+1)^{2/3} - n^{2/3})$$

Table S4: The values of the properties used in the calculation of the expected slope and intercept for the EB-AVBMC fitting procedure. Density values are coexistence densities.

Temperature (K)	ρ_{gas} (g/cm^3)	ρ_{liq} (g/cm^3)	γ (mN/m)	Sources[S4]
300	2.559×10^{-5}	0.99651	71.686	NIST
373	5.952×10^{-4}	0.95846	58.941	NIST

References

(S1) Hofer, T. S.; Wiedemair, M. J. Towards a dissociative SPC-like water model II. The impact of Lennard-Jones and Buckingham non-coulombic forces. *Physical Chemistry Chemical Physics* **2018**, *20*, 28523–28534.

(S2) Soper, A. The radial distribution functions of water and ice from 220 to 673 K and at pressures up to 400 MPa. *Chemical Physics* **2000**, *258*, 121–137.

(S3) Loeffler, T. D.; Chan, H.; Sasikumar, K.; Narayanan, B.; Cherukara, M. J.; Gray, S.; Sankaranarayanan, S. K. Teaching an old dog new tricks: Machine learning an improved TIP3P potential model for liquid–vapor phase phenomena. *The Journal of Physical Chemistry C* **2019**, *123*, 22643–22655.

(S4) Linstorm, P. NIST chemistry webbook, NIST standard reference database number 69. *J. Phys. Chem. Ref. Data, Monograph* **1998**, *9*, 1–1951.
